# Seagrass as a potential nutraceutical to decrease pro-inflammatory markers

**DOI:** 10.1186/s12906-024-04532-z

**Published:** 2024-07-10

**Authors:** Vani Mathakala, Tejaswini Ullakula, Uma Maheswari Devi Palempalli

**Affiliations:** Department of Applied Microbiology & Biochemistry, Sri Padmavati Mahila Visvavidyalayam (Women’s University, Tirupati, 517 502 A.P India

**Keywords:** Seagrass *Halophila beccarii*, RAW 264.7 macrophages, COX-2, Inducible nitric oxide synthase, Inflammatory cytokines

## Abstract

**Background:**

The Pro-inflammatory mediators such as prostaglandin E2, nitric oxide and TNF-α are the key players in the stimulation of the inflammatory responses. Thus, the pro-inflammatory mediators are considered to be potential targets for screening nutraceutical with anti-inflammatory activity.

**Methods:**

In this context, we explored the anti-inflammatory potency of seagrass extract with western blot (Bio-Rad) analysis by using LPS induced RAW macrophages as in-vitro models, western blot analysis, In-silico methods using Mastero 13.0 software.

**Results:**

The anti-inflammatory activity of Seagrass was demonstrated through down regulation of Pro-inflammatory markers such as Cyclooxygenase-2, induced Nitric oxide synthase and prostaglandin E synthase-1**.** The results were validated by docking the phytochemical constituents of seagrass namely Isocoumarin, Hexadecanoic acid, and Cis-9 Octadecenoic acid, 1,2 Benzene dicarboxylic acid and beta-sitosterol with TNF-alpha, COX-2, iNOS and PGES-1.

**Conclusion:**

The methanolic extract of seagrass *Halophila beccarii* is a potential nutraceutical agent for combating against inflammation with a significant anti-inflammatory activity.

## Introduction

Seagrasses are marine flowering plants distributed along the coasts of south and south-eastern Asia. Seagrass appears as meadows and fastens to the ocean by thick root system along with rhizomes and closely resembles the ecosystem of mangroves and coral reefs. More than fifteen species of seagrasses belonging to the family Hydrocharitaceae are identified in the pulicat lake the second largest salt lagoon, spanning around 750 sq.km across Andhra Pradesh and Tamil Nadu, India. Vani et al. [1] reported the distribution of seagrass *Halophila beccarii* from the estuarine environments of Pulicat Lake and Andaman and Nicobar Islands,. Currently, marine plants like seagrasses are gaining popularity due to their adaptation to the marine environment.

The qualitative and quantitative analysis of an organic extract of *H. beccarii* exhibited the presence of essential polyunsaturated fatty acids, polyphenols, flavonoids and proteins [1]. The phenols and flavonoids are active ingredients in several terrestrial plants and are reputed to play a part in the treatment of oxidative stress and inflammation [2].

Inflammation is associated with several chronic diseases like cancer, rheumatoid arthritis, cardiovascular and neurodegenerative disorders [3]. During the process of inflammation, macrophages play a specific role in the secretion of pro-inflammatory mediators such as prostaglandins and nitric oxide [4]. Lipopolysaccharide, one of the components of the Gram negative bacterial cell wall, is an efficient simulator in the secretion of pro-inflammatory cytokines like TNF-α and secondary mediators such as (NO), leukotrienes, prostaglandins (PGs) and super oxide anion from activated macrophages [5] Moreover, the COX-2 and iNOS are linked with the progression of inflammatory diseases [6]. Therefore, the down regulation of inflammatory enzymes and the inhibition of nitric oxide, PGE2 and TNF-α production are the hall mark targets to develop anti-inflammatory agents [7].

The proposed research paper with the hypothesis that the seagrass extract could be a potential anti-inflammatory agent and evaluates this potential through in-vitro analysis. The methanolic extract was selected because it is noted with bio active metabolites rich in unsaturated fatty acids, flavonoids and phenolic compounds. The seagrass *H. beccarii* contains a plethora of bio active compounds which was evaluated earlier through GC–MS analysis (Vani et al. 2023). Thus, the proposed research evaluates the synergistic action of these compounds, extracted from seagrass *H. beccarii*, in augmenting the anti-inflammatory effect through in-vitro analysis in LPS stimulated raw macrophages.

## Materials and methods

### Seagrass extract

The air dried powder (20 g) of seagrass was mixed with 200 mL of water, methanol and ethanol independently and maintained at room temperature for seven days. The mixtures were subjected to liquid–liquid extraction and rotary evaporation to obtain the crude extract for the experimental analysis. The preparation of crude extracts was obtained by mixing 20 g of powder with water (250 ml), methanol or ethanol solvent (250 ml) using Soxhlet apparatus for 72 h. To eliminate particulates, each volume of crude extracts were filtered individually through Whatman No. 41 filter paper. To get dry crude extracts, the particle-free crude extracts were completely evaporated using a rotary evaporator (Polylab, India) under reduced pressure. After completion of evaporation, the yield of aqueous extract (1.51 g), methanol crude extract (2.54 g) and ethanol crude extract (1.89 g) was calculated respectively.

### Anti-hemolytic assay of seagrass extract

Six varied concentrations of seagrass extracts (aqueous, ethanol and methanol) ranging from 50,100, 200, 300, 400 and 500 µg/mL in 0.1% DMSO were utilized in the HRBC membrane stabilization assay for detecting the in vitro anti-inflammatory activity and anti-protein denaturation activity [8]. Among the three extracts, methanol extract exhibited potential anti-inflammatory activity and it was chosen for further work.

### Toxicity analysis of seagrass extract

MTT(3-[4,5-dimethylthiazol-2-yl]-2,5 diphenyl tetrazolium bromide) assay was adopted to analyze the Cytotoxicity of seagrass extract with a concentration ranging from 100 µg to 500 µg/ml [9] by using RAW 264.7 macrophages. The percentage of cell viability was detected based on the formazan crystals formed by mitochondrial succinate dehydrogenase activity.

### Impact of seagrass extract on secretion of pro-inflammatory mediators

To analyze the effect of seagrass extract on inflammatory mediators, the RAW 264.7 macrophages induced with LPS were used as an *in-vitro* model. The RAW264.7 macrophages were cultured in a DMEM medium comprising FBS, 100 U/mL of penicillin and 10 µg/mL of Streptomycin and maintained at 37 °C in a 5% CO_2_ humidified atmosphere. After the confluence of growth, the cells were treated with the methanolic extract at different concentrations (20–100 µg) for 1 h before stimulation with LPS (1 µg/mL) for 24 h for the production of NO and TNF-α and only 4 h for the secretion of PGE2 [10]. After stimulation with LPS for 24 h, the cell-free culture medium was separated by centrifugation at 1000 G-force for 15 min and 50 µl of cell-free extract was used to determine the NO production by Griess reagent [4] and TNF-α by ELISA Kit as per the instructions of Bioscience (San Diego, CA, USA). The quantity of PGE2 released from endogenous arachidonic acid in the murine macrophages was measured with PGE2 assay ((Thermo Fische Scientific, Chennai, India)).

### Seagrass extract on the translational expression of pro-inflammatory enzymes

After 4 h and 24 h of stimulation with LPS, the cells were lysed and the extracted proteins were resolved on 10% SDS-PAGE to analyze the translational expression of COX-1,COX-2 and iNOS respectively. The protein bands were trans blotted on to nitrocellulose membrane for probing with anti-COX-1 and anti- COX-2 (1:1000), iNOS (1: 5,000) primary antibodies and subsequently for 1 h with anti-rabbit IgG (1:10,000). The bands were subjected to an ECL assay for visualization. The equal loading of cytoplasmic proteins was validated with glyceraldehyde-3-phosphate dehydrogenase..

### In-silico analysis for anti-inflammatory activity

#### Preparation of inflammatory proteins

The crystalline structure of the TNF-alpha, COX-1, COX-2, iNOS and mPEGS1 were obtained from protein data bank (PDB ID: 3GIO, 3N8Y, 1PXX, 4NOS and 3DWW). The protein structures were prepared by remodeling the co-crystallized structures and water molecules.

### Preparation of seagrass bio active compounds

The compounds of the seagrass extract such as Isocoumarin, Docosanoic acid, hexadecanoic acid, Cis-9 Oct-decanoicacid, Triacontane, 1,2 Benzene dicarboxylic acid, 2,6 Tetracosapentamethyl eicosapetane, beta-sitosterol and standard drugs ( Diclofenac sodium salt, Ethyl Isothiourea) which are characterized through GC–MS (SHIMADZU) analysis [11] were selected as ligands and their structures were derived in SDF format from the pubchem database [12].

### Interaction of inflammatory proteins with bio active ligands

Mastero 13.O was employed for predicting interaction of Docosanoic acid (Behenic acid), Isocoumarin, Hexadecanoic acid, Cis-9Octadecenoic acid, Triacontane, 1,2 Benzene dicarboxylic acid, 2,6,10,14,18-Tetracosa pentamethyl eicosapentaene, Beta-sitosterol with various target proteins such as COX-1, COX-2, iNOS and mPEGS1 [13, 14]. The grid box of the target proteins was generated for flexible docking with the ligands and also to enhance the binding of bio active ligands in the active pocket of target proteins by minimizing the torsion angles [15].

#### Statistical analysis

Analysis of variance (ANOVA) and Duncan's multiple comparison test (DMCT) was performed to calculate the statistical significance of the experimental data. Statistical significance is denoted by an asterisk (*) when *p* values are *p < 0.03 to 0.05, **p < 0.001, ***p < 0.0001 and ****p < 0.00001.

## Results and discussion

Inflammation is the basic mechanism triggered by toxic stimuli and several pathogens [16]. During infection by gram negative bacteria, the microbial antigen, namely LPS stimulates the macrophages and leads to the induction of acute or chronic inflammatory diseases. Hence, murine macrophages stimulated with LPS are commonly utilized to characterize anti-inflammatory agents targeting pro-inflammatory enzymes and cytokines [17, 18]. As per the current literature, there is a need to explore the potential effects of marine phytochemicals against many inflammatory mediated human diseases [19]. The current data reveals the anti-inflammatory potential of *H. beccarii* collected from Pulicat Lake, India.

### Anti-inflammatory activity of seagrass extract

The Anti-inflammatory activity of seagrass was analyzed by HRBC membrane stabilization and anti-protein denaturation assay. HRBC membrane stabilization by seagrass extract against the degrading effect induced by heat was tabulated (Table 1). Among the three extracts, methanol extract showed significant inhibition of hemolysis with IC_50_ at 100 µg/mL in comparison with standard Disperin. Inhibition of protein denaturation by various concentrations of seagrass extract (50–500 µg/mL) and diclofenac was represented in Table 2. In correlation with standard NSAID diclofenac, methanolic extract of seagrass exhibited significant anti-inflammatory activity.

**Table 1:** Stabilization of HRBC membrane by seagrass extract

Concentration µg/mL	% inhibition of Hemolysis			
	AE	EE	ME	Diclofenac
50	15 ± 1.81^a^	25 ± 1.01^a^	45 ± 4.11^a^	65 ± 3.22^b^
100	24 ± 0.81^b^	36 ± 2.11^b^	55 ± 3.31^a^	76 ± 2.51^c^
200	35 ± 1.51^a^	44 ± 3.01^a^	62 ± 2.41^c^	84 ± 1.21^a^
300	40 ± 2.01^c^	50 ± 2.51^c^	70 ± 3.21^a^	91 ± 3.25^c^
400	45 ± 1.71^a^	57 ± 4.01^c^	76 ± 2.21^b^	94 ± 4.24^c^
500	55 ± 1.41^c^	65 ± 3.01^a^	85 ± 4.11^a^	98 ± 3.20^a^
IC/50 Value µg/mL	555 ± 1.81	454 ± 5.01	100 ± 4.12	45 ± 2.21^b^

*AE* Aqueous extract, *EE *Ethanol extract, *ME *Methanol extract of seagrass *Halophila beccarii*)

Values are given as mean ± SEM, values with the same superscript indicate no difference in value (*p* *≤* 0.05)

**Table 2:** Effect of seagrass extract on the inhibition of Protein denaturation

Concentration µg/mL	% inhibition of Protein denaturation			
	AE	EE	ME	Disperin
50	12 ± 0.81^a^	18 ± 1.01^a^	40 ± 4.01^a^	60 ± 2.82^b^
100	20 ± 1.61^b^	30 ± 2.01^b^	45 ± 3.11^a^	65 ± 2.21^c^
200	30 ± 1.71^a^	32 ± 2.91^a^	52 ± 2.21^c^	70 ± 1.11^a^
300	32 ± 2.03^c^	40 ± 2.31^c^	57 ± 3.11^a^	80 ± 3.05^c^
400	44 ± 1.72^a^	47 ± 3.81^c^	64 ± 2.11^b^	85 ± 4.04^c^
500	45 ± 1.31^c^	55 ± 3.11^a^	75 ± 2.81^a^	90 ± 2.20^a^
IC/50 Value µg/mL	555 ± 3.71	454 ± 6.01	185 ± 3.12	50 ± 3.21^b^

*AE* Aqueous extract, *EE* Ethanol extract, *ME* Methanol extract of seagrass *Halophila beccarii*

Values are given as mean ± SEM, values with the same superscript indicate no difference in value (*p* ≤ 0.05)

### Chemical profile of seagrass extract

**Fig. 1:**
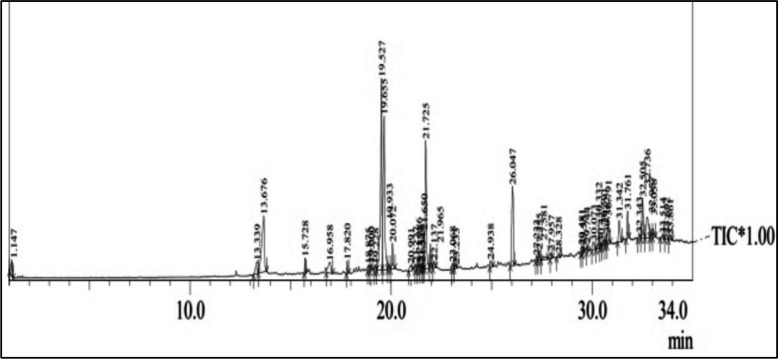
GC–MS Chromatogram of Seagrass methanol extract

We reported the presence of isocoumarin, Docosanoic acid, hexadecanoic acid, cis-9 octa decanoic acid, Triacontane, 1,2 Benzene dicarboxylic acid, 2,6,10,14,18-Tetracosapentamethyl eicosapetane, beta-sitosterol in the methanolic extract of seagrass through GC–MS analysis (Fig. 1) [11]. Several studies have indicated the role of unsaturated fatty acids in the down regulation of pro-inflammatory mediators, antimicrobial effect of Docosanoic acid [20] and behenic acid (Table 3) in maintaining the tissue integrity [21] and anti-hyperlipidemic activity and regulation of glucose transport by methanolic extract of seagrass [22].

**Table 3:** The chemical profile of seagrass methanol extract [1]

S.No	RT	Compound Name	Molecular Structure	MW
1	1.14	Isocoumarin		146
2	13.6	Docosanoic acid (Behenic acid)		340.6
3	19.52	Hexadecanoic acid		256.4
4	19.65	Cis-9 Octadecanoic acid		282.0
5	19.93	Triacontane,		422.8
6	21.72	1,2Benzene dicarboxylic acid		152.1
7	26.04	2,6,10,14,18-Tetracosapentamethyleicosapentaene		342.6
8	32.5	Beta-sitosterol		2456.7

*RT* Retention time *MW *Molecular weight

### Cytoprotective effect of seagrass extract on macrophages

**Fig. 2:**
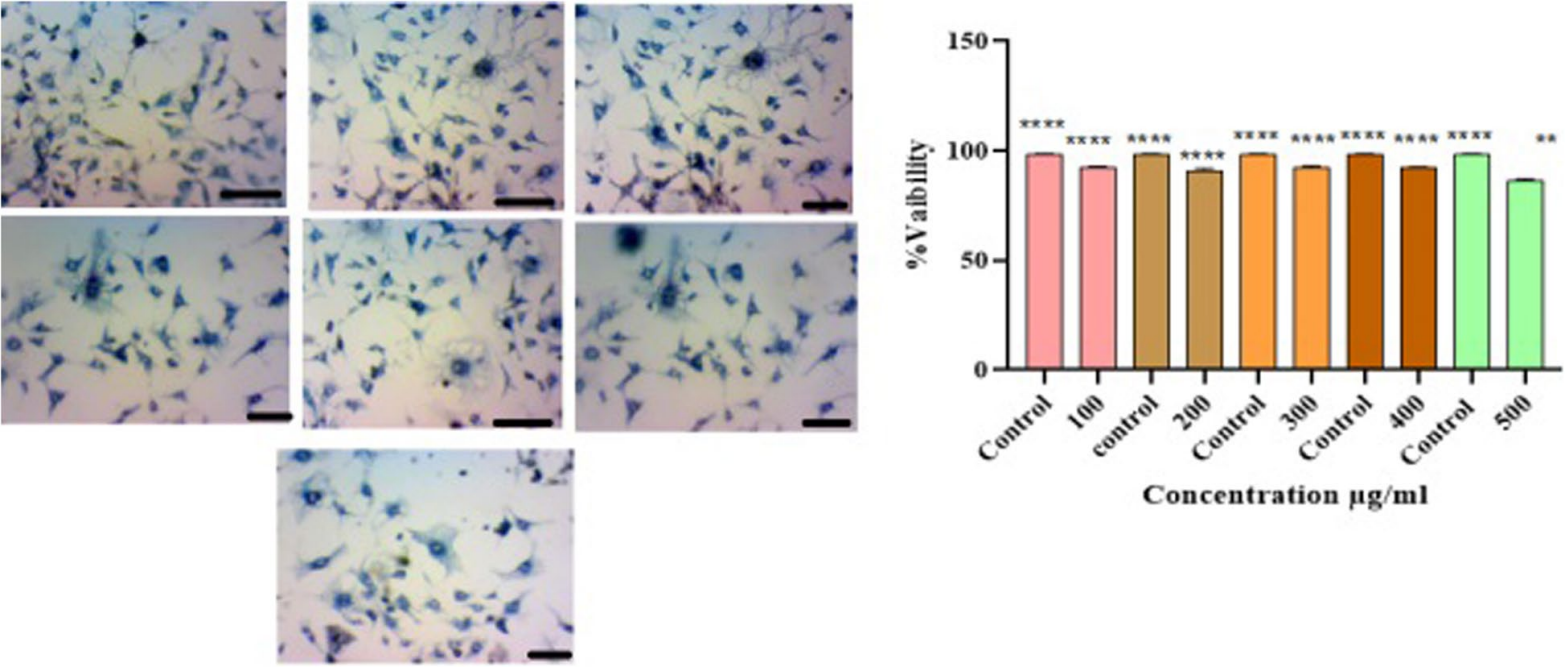
Effect of seagrass extract (100 µg/ml to 500 µg/ml) on proliferation of Raw 264.7 Cells. Data represents the mean ± SEM of three independent experiments with significance of 1% level (*p* ≤ 0.0001)

The MTT assay has been widely used to assess mitochondrial respiration and cell viability [23]. Further, there is no change in the morphology and shape of the macrophages even at the higher concentration of methanolic extract which represents the cytoprotective effect of seagrass extract (Fig. 2). The methanol extract of seagrass was found to be nontoxic and the percentage proliferation of treated cells was on par with the control cells of RAW 264.7 macrophages. where as seagrass *Thalassodendron ciliatum* from red sea exhibited Cytoprotective activity on treatment with MCF-7 cells [24]

### Inhibition of LPS-induced nitric oxide production

**Fig. 3:**
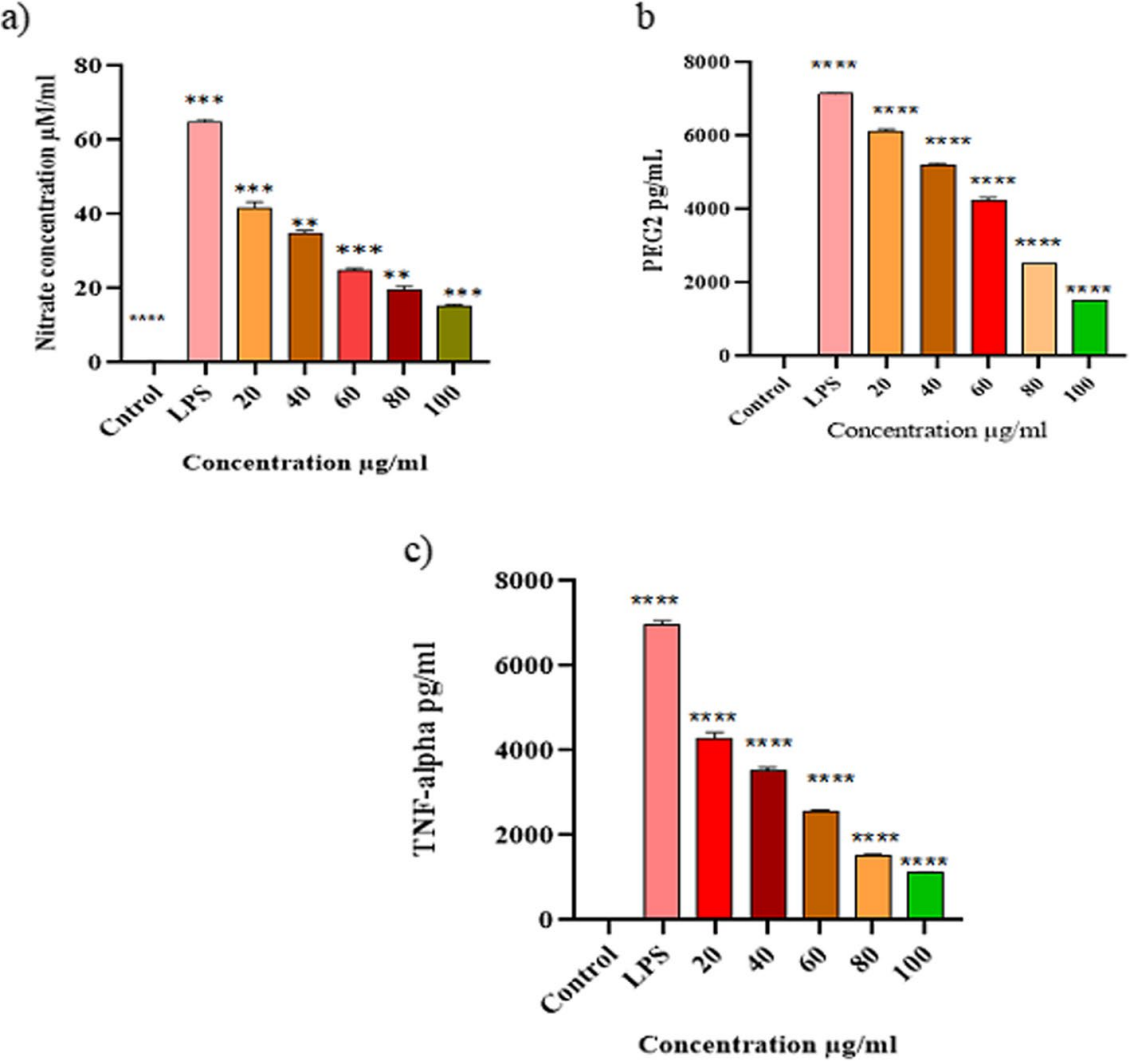
**a** Influence of seagrass extract on the extracellular secretion of NO in LPS stimulated Raw 264.7 macrophages. **b** and **c** The effect of seagrass extract on the expression of PGE2 and TNF-α in LPS stimulated RAW 264.7 macrophage cells. Values are expressed as mean ± SEM of three independent experiments, statistical significance 1% level (*p* ≤ 0.0001)

The nitrite concentration, an indicator of nitric oxide production, was estimated by Griess assay by using culture supernatant of LPS stimulated cells in the presence of seagrass extract. In LPS stimulated cells, the nitric oxide levels were remarkably high (65.21 ± 2.35 μM) compared to control macrophages wherein the level of NO was 3.83 ± 1.25 μM (*P* < 0.05). On the other hand, the seagrass extract showed strong NO inhibition with the lowest IC50 of 45 µg/ml. The secretion of nitrite was drastically lowered with gradual increase in the dose of methanol extract of seagrass (Fig. 3a). LPS stimulated macrophages release excessive nitric oxide due to the degradation of arginine and the production of nitric oxide is regulated by iNOS [25].

### PGE2 & TNF-alpha production

During the process of acute and chronic inflammation, macrophages produce inflammatory cytokines like TNF- α and PGE2 to protect the body from bacterial infection and macrophages as reservoir source for the secretion of PGE2 [26]. The effect of the methanolic extract on PGE2and TNF-α secretion in the culture supernatant of macrophages cells was measured through immunoassay [27]. The cells treated with LPS exhibited a significant activation of PGE2 & TNF-α production. The assay demonstrated dose-dependent inhibition of TNF- α & PGE2 secretion by seagrass extract at 20, 40, 60, 80, 100 µg/mL (Fig. 3b and c). Our data demonstrates that seagrass extract could specifically prevents the production of PGE2 stimulated by the LPS. Thus, seagrass extract that attenuate the production of inflammatory mediators have had a healing effect on many inflammation-related diseases [3].

### Down regulation of COX-2 and iNOS expression by seagrass extract

**Fig. 4:**
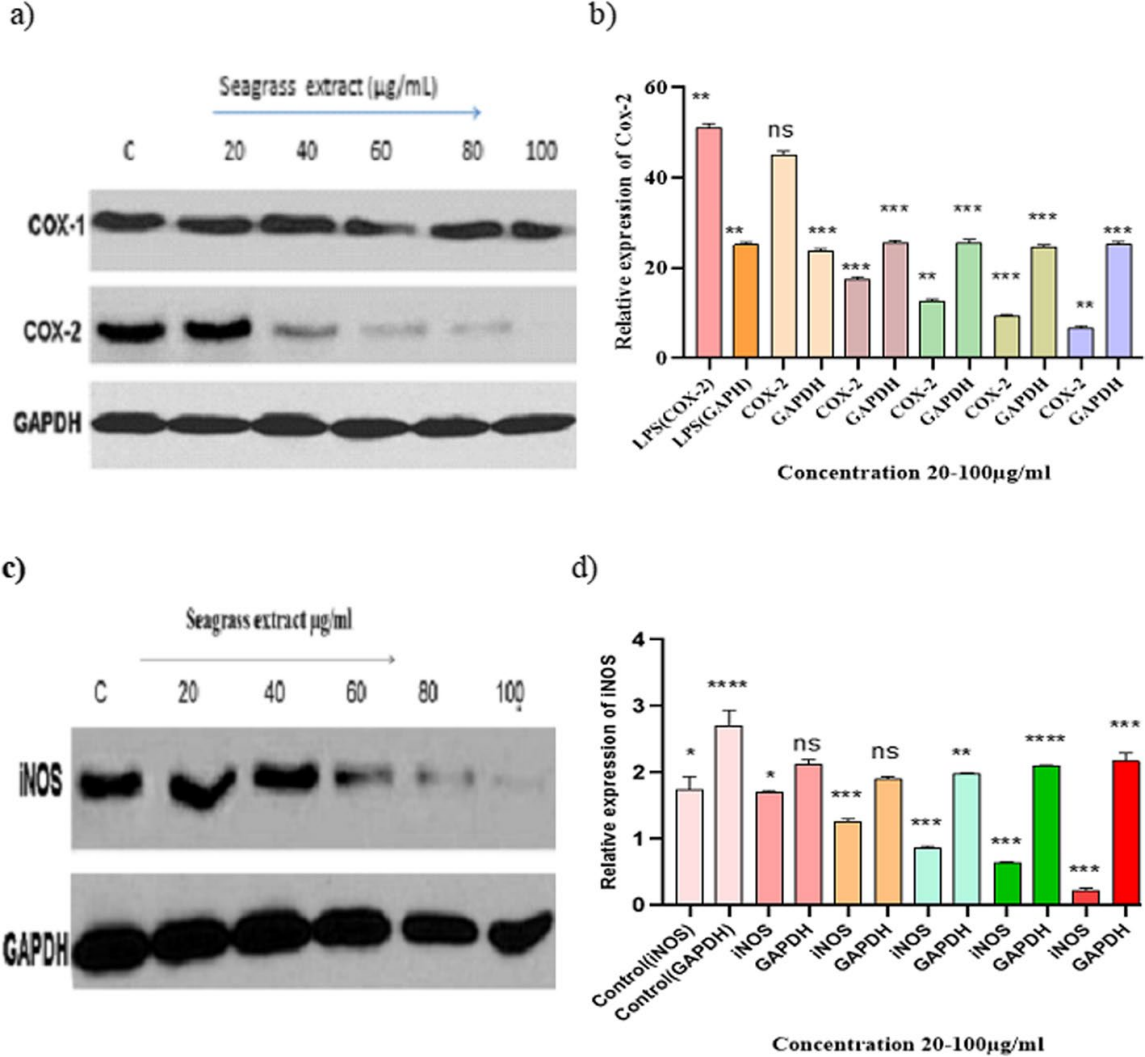
**a** Translational expression of COX-1 and COX-2 in macrophages treated with seagrass extract. **b** The densitometry data of COX-2 protein expression normalized with loading control GAPDH. **c** Expression profile of iNOS in macrophages treated with seagrass extract. **d** The densitometric data of iNOS expression normalized with loading control GAPDH. *P* ≤ 0.0001, Data is represented as mean ± SD values from 3 independent experiments with each experiment done in triplicate

The cells like macrophages, endothelial cells and monocytes induce the production of COX-2 enzyme on exposure to various stimuli [28]. In addition, the macrophages stimulated with LPS secrete excess inflammatory enzymes, particularly COX-2 (Fig. 4a and b), the key enzyme involved in the synthesis of PGE2 [29]. The impact of seagrass extract on the expression of COX proteins was investigated by Western blot analysis. As per the results presented in Fig. 4a, COX-1 protein levels were constitutive in all the samples. GAPDH was used as a loading control. On the contrary, the downward profile of COX-2 protein was noticed in macrophages treated with seagrass extract in dose-dependent cascade with an IC50 value of 60 μg/mL. Cyclooxygenases exist in three isoforms COX-1, COX-2 and COX-3. Among the three isoforms, the COX-1 performs a housekeeping function due to its constitutive nature [30] and the inducible isoform COX-2 catalyzes the synthesis of PGE2, the inflammatory mediator which contributes to the pathogenesis of several inflammatory disorders [31].

Endothelial nitric oxide synthase (eNOS), neuronal nitric oxide synthase (nNOS) and inducible nitric oxide synthase iNOS are three different forms of nitric oxide synthases. Among the three isoforms, iNOS is induced in response to LPS in macrophages. We also analyzed the effect of seagrass extract on the translational expression of iNOS in LPS-stimulated RAW 264.7 cells. As represented in Fig. 4c and d, LPS increased the expression of iNOS in macrophages, however, the expression was significantly lowered on treatment with seagrass extract in a concentration dependent manner.

### In-silico analysis for the identification of anti-inflammatory compounds

The seagrass compounds such as Isocoumarin, 1, 2 benzene dicarboxylic acids, Cis-9 Octadecenoic 2,6,10,14,18-Tetracosapentamethyl eicosapentaene acid, Hexadecenoic acid, Triacontane, Beta sitosterol, and Docosanoic acid (Behenic acid) were selected as ligands to analyze the interaction with inflammatory markers such as TNF-alpha, COX-2, PGE2, and iNOS and drugs such as diclofenac sodium salt and ethyl isothiourea were used as a standard anti-inflammatory agents in Mastero 13.O (Table 4).

**Table 4:** Docking energy values and interactions of seagrass bio active metabolites and standard Anti-inflammatory agents with TNF-alpha, COX-2, mPGES1 and iNOS proteins

Name of the ligands	Target protein	Active site amino acids	H-bond length	Docking energy
Diclofenac sodium salt(standard)	TNF-alpha	Tyr-119, Ser-60	1.64, 1.72	-7.451
1,2 Benzene dicarboxylic acid		Leu-57, Gln-61	1.79, 1.69	-6.879
Isocoumarin		Ser-530	1.99	-5.431
Diclofenac sodium salt	COX-2	Tyr-385, Ser-530	1.67, 1.82	-9.674
1,2 Benzene dicarboxylic acid		Ser-530, Try-385	1.88, 1.86	-7.902
Isocoumarin		Ser-530, Trp-387	2.23, 5.37	-7.359
Diclofenac sodium salt	mPGES1	Arg-110, Arg-126	1.85, 3.32	-5.338
Isocoumarin		Arg-126, Arg-110	2.17, 4.88	-4.759
1,2 Benzene dicarboxylic acid		Arg-126, Arg-110	1.90, 2.23	-4.701
1,2 Benzene dicarboxylic acid	iNOS	Trp-372, Glu-377	1.86, 1.57	-5.351
Ethyl Isothiourea (standard)		Tyr-194	4.11	-6.455
Isocoumarin		Arg-199, Tyr-491	2.74, 2.32	-5.389

**Fig. 5:**
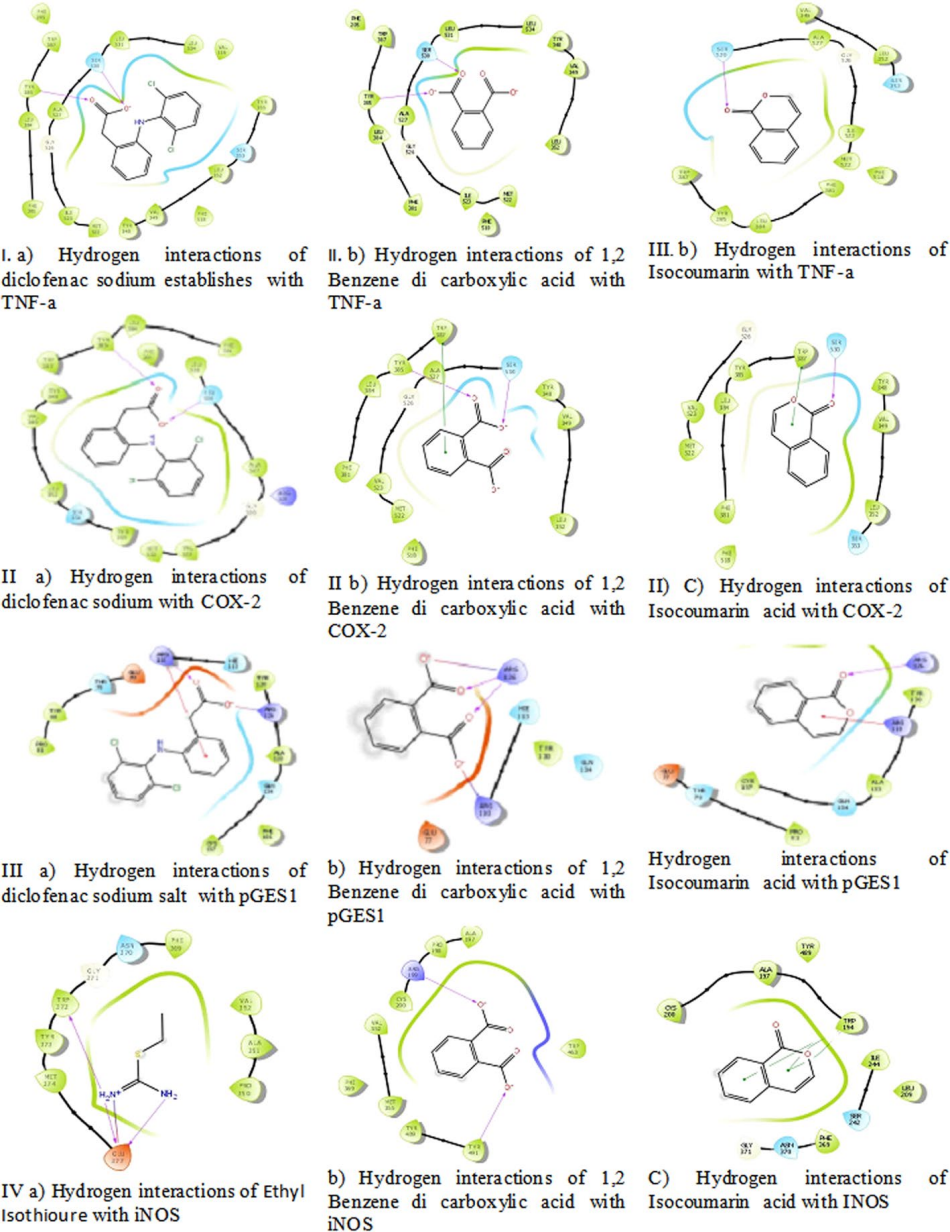
I. Docked pose of site of TNF-alpha with seagrass bio active metabolites and standard drug. II. Molecular docking of COX-2 active sites with ligands. III. Docking Interaction of mPGES1 with seagrass bio active ligands. IV. Docking pose of site of iNOS with seagrass metabolites

Based on the docking scores of seagrass compounds, the isocoumarin and 1,2 benzene dicarboxylic acid with TNF was exhibited hydrogen interaction with Leu-57, Gln-61 and Ser-530 respectively with docking energy comparable to diclofenac sodium salt (Fig. 5 Ia, Ib, Ic). As per the earlier reports [26] Tyr-385, Ser-530 are the catalytic amino acid residues of COX-2. Both standard and seagrass compounds showed similar hydrogen bond pattern with catalytic amino acids. Thus the isocoumarin and 1,2 benzene dicarboxylic acids are key players of seagrass in the translational inhibition of COX-2 enzyme (Fig. 5 IIa, IIb, IIc).

The anti inflammatory activity of isocoumarin and 1,2 benzene dicarboxylic acid were further validated by docking with mPGES1 [32] reported the up-regulation of mPGES1 by pro- inflammatory stimuli and the role of glucocorticoids in the down regulation of mPGES1. Currently, mPGES1, is gaining attention as a key target for anti-inflammatory drugs due to its expression in diseased tissue [33] and elevated levels in several types of cancers. Seagrass compounds extend hydrogen interaction with two catalytic amino acids, namely Arg-110, and Arg-126 and exhibit mimicking characteristics both in terms of hydrogen bond formation and docking affinity (Fig. 5 IIIa, IIIb, IIIc). We therefore confirm that seagrass compounds blocks the production of PGE2 through the inhibition of microsomal PGES1. To further test our hypothesis, the compounds were docked with another inflammatory enzyme iNOS. As per the data shown, both the compounds were effective against iNOS and showed strong interaction with aromatic amino acids (Fig. 5 IVa, IVb, IVc).

## Conclusion

The methanol extract of seagrass *H. beccarii* was found to be nutraceutical with an anti-inflammatory property demonstrated by the reduction in the production of pro- inflammatory markers like TNF-alpha, PGE-2, COX-2 and. The specific composition for the anti-inflammatory effect was identified through in-silico analysis by docking as Isocoumarin and Benzene dicarboxylic acid in the seagrass extract showed a significant potential as evidenced by strong binding and formation of stable complexes with COX-2, TNF-alpha and other pro-inflammatory markers. The result showed a desired effect of the compounds with an anti-inflammatory property equivalent to widely used drugs like Diclofenac sodium. However, the in-vivo/human analysis for the extract was not within the purview of the study and hence the potency of the extract could not be quantified concretely in humans. The desired effect exhibited by the seagrass extract, provides an untapped market for the food industry to explore and develop nutraceuticals.

## Data Availability

The entire data is available in the manuscript as well as in the research square.

## References

[1] Vani M, Murthy SDS, Uma Maheswari Devi P. Phytochemicals and *in-vitro* Antioxidant activity of *Halophila Beccarii*. Int J Pharm Sci Res. 2019;10(3):1347–53.

[2] Luthria DL (2006). Significance of sample preparation in developing analytical methodologies for accurate estimation of bioactive compounds in functional foods. J Sci Food Agric.

[3] Lee J, Aoki T, Thumkeo D, Siriwach R, Yao C, Narumiya S (2019). T cell-intrinsic prostaglandin E2-EP2/EP4 signaling is critical in pathogenic TH17 cell-driven inflammation. J Allergy Clin Immunol.

[4] Heo SJ, Yoon WJ, Kim KN (2012). Anti-inflammatory effect of fucoxanthin derivatives isolated from Sargassum siliquastrum in lipopolysaccharide-stimulated RAW 264.7 macrophage. Food Chem Toxicol..

[5] Kil-Nam Kim KK, Soo-Jin H, Weon-Jong Y, Sung-Myung K, Ginnae A, Tae-Hoo Yi, You-Jin J. Fucoxanthin inhibits the inflammatory response by suppressing the activation of NF-κB and MAPKs in lipopolysaccharide-induced RAW 264.7 macrophages. Eur J Pharmacol. 2010;649(1–3):369–75. 10.1016/j.ejphar.2010.09.032 20868674

[6] Vunta H, Davis F, Palempalli UD, Bhat D, Arner RJ, Thompson JT (2007). The anti-inflammatory effects of selenium are mediated through 15-deoxy-Δ12, 14-prostaglandin J2 in macrophages. Int J Biol Chem.

[7] Simmons DL, Botting RM, Hla T (2004). Cyclooxygenase isozymes: the biology of prostaglandin synthesis and inhibition. Pharmacol Rev.

[8] Williams DE, Austin P, Diaz-Marrero AR, Soest RV, Matainaho T, Roskelley CD, Roberge M, Andersen RJ (2005). Neopetrosiamides, peptides from the marines ponge Neopetrosia sp. that inhibit amoeboid invasion by human tumor cells. Org Lett..

[9] Uma maheshwari devi P, Ujjawal G, Parisa K, Hema V, Ryan JA, Vivek N (2009). Gambogic acid covalently modifies IkB kinase-β subunit to mediate suppression of lipopolysaccharide-induced activation of NF-κB in macrophages. Biochem J..

[10] Chou TC, Fu E, Shen EC (2003). Chitosan inhibits prostaglandin E2 formation and cyclooxygenase-2 induction in lipopolysaccharide-treated RAW 264.7 macrophages. Biochem Biophys Res Commun..

[11] Vani M, Muni Kesavulu M 2, Uma Maheswari Devi P. Halophila beccarii extract ameliorate glucose uptake in 3T3-L1 adipocyte cells and improves glucose homeostasis in streptozotocin-induced diabetic rats. Heliyon.2022; 8(8):e10252. 10.1016/j.heliyon.2022.e10252 PMC9420365 36042748

[12] Wang R, Feng X, Zhu K, Zhao X, Suo H (2016). Preventive activity of banana peelpolyphenols on CCl4-induced experimental hepatic injury in Kunming mice. Exp Ther Med.

[13] Brogi S, Quimque MT, Notarte KI, Africa JG, Hernandez JB, Tan SM, Calderone V (2022). Macabeo, AP (2022) Virtual Combinatorial Library Screening of Quinadoline B erivatives against SARS-CoV-2 RNA-Dependent RNA Polymerase. Computation.

[14] de Leon VNO, Manzano JAH, Pilapil DYH, Fernandez RAT, Ching JKAR, Quimque MTJ, Agbay JCM (2021). Anti-HIV reverse transcriptase plant polyphenolic natural products with in silico inhibitory properties on seven non-structural proteins vital in SARS-CoV-2 pathogenesis. J Genet Eng Biotechnol.

[15] Santos J, Quimque MT, Liman RA, Agbay JC, Macabeo APG, Corpuz JA, Wang YM, Lu TT, Lin CH, Villaflores OB (2021). Computational and xperimental Assessments of Magnolol as a Neuroprotective Agent and Utilization of UiO-66(Zr) as Its Drug Delivery System. ACS omega 6..

[16] Sungeun A, Priyanka CA, Verónica Y, Shakina K, Yu-Jin Kim (2017). Gold nanoparticles synthesized using Panax ginseng leaves suppress inflammatory- mediators production via blockade of NF-κB activation in macrophages. Artif Cells Nanomed Biotechnol..

[17] Zhong Y, Chiou YS, Pan MH, Shahidi F (2012). Anti-inflammatory activity of lipophilic epigallocatechin gallate (EGCG) derivatives in LPS-stimulated murine macrophages. Food Chem.

[18] Kwon DH, Cha HJ, Choi EO, Leem SH, Kim GY, Moon SK, Chang YC (2017). Schisandrin A suppresses lipopolysaccharide-induced inflammation and oxidative stress in RAW 264.7 macrophages by suppressing the NF-κB, MAPKs and PI3K/Akt pathways and activating Nrf2/HO-1 signaling. Int J Mol Med..

[19] Recio MC, Andujar I (2012). Rios J (2012) Anti-inflammatory agents from plants: Progress and potential. Curr Med Chem.

[20] Spindola HM, Servat L, Denny C, Rodrigues RAF, Eberlin MN, Cabral E, Sousa IMO, Tamashiro JY, Carvalho JE, Foglio MA (2010). Antinociceptive effect of geranylgeraniol and 6α, 7β-dihydroxyvouacapan-17β-oate methyl ester isolated from Pterodon pubescens Benth. BMC Pharmacol.

[21] Campia I, Lussiana C, Pescarmona G, Ghigo D, Bosia A, Riganti C (2009). Geranylgeraniol prevents the cytotoxic effects of mevastatin in THP-1 cells, without decreasing the beneficial effects on cholesterol synthesis. British J Pharmacol.

[22] Vani M, Devi PU (2020). Seagrass in the Control of Hyperglycemic and Hyperlipidemic States of Streptozotocin Induced Diabetic Rats. Pharmacogn J..

[23] Navya A, Rayalu DJ, Maheswari Devi PU (2011). Docking studies on xanthones of mangosteen as cox-2 inhibitors. Int J Appl Biol Pharm.

[24] Abdelhameed RF, Ibrahim AK, Yamada K, Ahmed SA (2018). Correction to: Cytotoxic and anti-inflammatory compounds from Red Sea grass Thalassodendron ciliatum. Med Chem Res.

[25] Wink DA, Harry BH, Robert YSC, Christopher HS, Wilmarie FS, Michael PV, Lisa AR, Carol AC (2011). Nitric oxide and redox mechanisms in the immune response. J Leukoc Biol.

[26] Navya A, Rashmi H, Vasavi T, Palempalli UM (2014). Modulation Of Pro-Inflammatory Genes By α-Mangostin From Garcinia mangostana. Int J Pharm Sci..

[27] Muniandy K, Gothai S, Badran KMH, Kumar SS, Esa NM, Arulselvan P. Suppression of proinflammatory Cytokines and Mediators in LPS-Induced RAW 264.7 Macrophages by Stem Extract of Alternanthera sessilis via the Inhibition of the NF-kB Pathway. J Immunol Res. 2018; 2018(1):1–12. 10.1155/2018/3430684 PMC6093060 30155492

[28] Silva Oliveira ID, Oliveira Cardoso FD, Moragas Tellis CJ, Socorro dos Santos Chagas MD, Silva Calabrese KD (2019). ernonia polysphaera Baker: Anti-inflammatory activity in vivo and inhibitory effect in LPS-stimulated RAW 264.7 cell. Plos One..

[29] Zidar N, Odar K, Glavaˇc D, Jerše M, Zupanc T, Štajer D (2008). Cyclooxygenase in normal human tissues is COX-1 really a constitutive isoform, and COX-2 an inducible isoform?. J Cell Mol Med.

[30] Shin WB, Dong X, Kim YS, Park JS, Kim SJ, Go EA, Kim EK, Park PJ (2019). Anti-inflammatory E ects of Batillaria multiform is Water Extracts via NF-êB and MAPK Signaling Pathways in LPS-Induced RAW 264.7. Cells. Adv Exp Med Biol..

[31] Seung Hwa B, Tamina P, Myung-Gyun K, Daeui P (2020). Anti-Inflammatory Activity and ROS Regulation Effect of Sinapaldehyde in LPS-Stimulated RAW 264.7 Macrophages. Molecules..

[32] Murakami M, Kudo I (2006). Prostaglandin E Synthase: A Novel Drug Target for Inflammation and Cancer. Curr Pharm Des.

[33] Jiang X, Renkema H, Pennings B, Pecheritsyna S, Schoeman JC, Hankemeier T, Smeitink J (2021). Mechanism of action and potential applications of selective inhibition of microsomal prostaglandin E synthase-1-mediated PGE2 biosynthesis by sonlicromanol’s metabolite KH176m. Sci Rep..

